# An *ex vivo* biomechanical study of equine neonatal rib fracture repair by different fixation methods

**DOI:** 10.3389/fvets.2026.1854194

**Published:** 2026-07-15

**Authors:** Kayla Newell, Jenna Bayne, Fred Caldwell, Robert Cole, Ramsis Farag, David Redden, Jennifer Janes, Lindsey Boone

**Affiliations:** 1 Department of Clinical Sciences, College of Veterinary Medicine, Auburn University, Auburn, AL, United States; 2 Department of Polymer and Fiber Engineering, Samuel Ginn College of Engineering, Auburn University, Auburn, AL, United States; 3 Department of Textile Engineering, Mansoura University, Mansoura, Egypt; 4 Department of Biomedical Affairs and Research, Edward Via College of Osteopathic Medicine, Auburn, AL, United States; 5 Department of Veterinary Science, University of Kentucky Veterinary Diagnostic Laboratory, University of Kentucky, Lexington, KY, United States

**Keywords:** equine, modified reconstruction plate, nylon cable tie, rib clip, rib fracture, string of pearls plate

## Abstract

**Introduction:**

Rib fractures result in significant morbidity and mortality of equine neonates. Various methods to repair these fractures have been described but biomechanical testing in immature equine neonatal bone has not been performed. The study objective was to compare strength, stiffness, and mode of failure for four different methods of equine neonatal rib fracture repair.

**Methods:**

Ribs were randomly allocated to one of four repair groups: nylon cable tie (NCT), modified reconstruction plate (MRP), string of pearls plate (SOP), or rib clip (RC). Ribs were fractured under three-point bending to induce a simple, transverse fracture. The fracture was reduced and repaired according to the assigned repair group, then tested to failure under single-cycle four-point bending. Strength (maximum force, F_max_, and force at failure, F_fail_) and mode of failure were recorded. Stiffness (N/mm^2^) was calculated. Data was analyzed using linear mixed models.

**Results:**

In intact ribs, no significant differences in F_max_ (*p* = 0.46), F_fail_ (*p* = 0.35), or stiffness (*p* = 0.90) were observed between groups. Among repair groups, F_max_, F_fail_, and stiffness differed significantly (*p* < 0.0001). NCT repairs had significantly lower F_max_ and F_fail_ than MRP and SOP, while MRP and SOP had greater stiffness compared to NCT and RC. NCT and RC groups failed at the original fracture site, whereas MRP and SOP failed mostly due to screw pullout.

**Conclusion:**

Rigid fixation with MRP or SOP provides stronger fracture repair than NCT or RC when equine neonatal ribs are tested under single-cycle load to failure. Further investigation of both rigid and non-rigid repair methods with cyclic loading is warranted.

## 1 Introduction

Rib fractures account for 37% of all life-threatening fractures in foals, with approximately 21% of all foals affected, and mortality reported in up to 48% of these foals (1–3, 27). Fracture occurs more commonly in foals born to primiparous mares and/or mares with dystocia that require birthing assistance. Fracture usually occurs at or just dorsal to the costochondral junction of ribs 3–8 which overlie vital organs, and when displaced may result in severe, life-threatening injury such as pulmonary contusion or laceration of intercostal vessels, pleura, diaphragm, lung or heart, leading to hemorrhage, hemothorax, pneumothorax, diaphragmatic herniation, or even death (2). Up to 65% of rib fractures presenting to a referral hospital have moderate displacement, necessitating prompt diagnosis and treatment (4).

Conservative treatment with stall rest and pain management is commonly performed in foals; however, surgical stabilization of rib fractures (SSRF) is recommended for cranial ribs with or without displacement and foals with respiratory compromise (28). SSRF aims to restore stability to the chest wall, reducing further risk of injury to vital, intra-thoracic structures. In human medicine, SSRF has been associated with improved respiratory function, reduced pulmonary complications, reduced pain, decreased hospitalization, and costs compared to conservative management (5, 6). In infants, rib fracture occurs commonly due to non-accidental injury, with birthing trauma accounting for less than 8% of infants with rib fractures (7, 29). SSRF of the infant or even pediatric patients is often not pursued due to concern for disruption of normal rib growth and subsequent asymmetry of the chest wall. Despite differing case selection for SSRF between humans and foals, reports support repair of unstable rib fractures.

SSRF presents unique biomechanical challenges for fixation due to rib curvature, variable size, and continuous cyclical loading during respiration. The ideal repair method would be stable, histocompatible, minimize stress shielding, avoid neurovascular bundle entrapment, and be easily applied, minimizing surgical trauma to the patient. Fracture of the neonatal rib presents even more challenge due to the bone’s rapidly changing micro and macrostructure during growth placing unique and different biomechanical constraints on methods of repair compared to those used for mature bone (18).

Different repair methods have been employed in both human and veterinary medicine for rib fracture repair, including non-rigid and rigid fixation methods. Non-rigid fixation methods are relatively inexpensive and can be applied rapidly but may allow more movement at the fracture, leading to recurrence of fracture. Rigid fixation methods, including reconstruction and locking plate systems, provide greater stability to the repaired construct but are associated with implant-related complications such as screw loosening and/or pullout or decreased patient comfort (30). In human SSRF, rigid fixation methods are associated with hardware failure and implant-related discomfort, yet rigid fixation remains the most common repair method. Current SSRF repair constructs include cerclage with suture or wire with or without external splinting (mesh or K wires or pins); metal (titanium or stainless steel) or bioresorbable plates applied to the bone via screws (cortical or locking screws), clips or staples; clip fixators; intra-medullary fixators; U-plates and strut or Nuss bars (8). Several of these described techniques have been used and modified for equine SSRF. Methods include suturing, cerclage wire alone or in combination with rigid implants (pins or plates secured to the outer surface of the rib), Securos cranial cruciate ligament repair system™, nylon cable tie, and modified reconstruction plate applied with screws and/or cerclage wire (9, 10). For SSRF in foals, it is vital that the method chosen is applied quickly and easily while supporting fracture healing with little to no implant-related complications.

Bellezzo et al. (10) first described reconstruction plating of equine neonatal rib fractures, with modifications to the technique still commonly used (15, 28). Reconstruction plating requires proper plate contouring and positional cortical screws that have limited purchase in immature bone, leading to screw pull-out. Current recommendations use a 2.7 mm reconstruction plate with holes, re-drilled to accommodate a larger, 3.5 mm cortical screw for greater purchase in the bone. Cerclage wire is also often placed to further stabilize the plate to the bone and prevent screw pull-out. This is one of the most commonly used method of SSRF in equine neonates.

Locking plate use has only been described for adult equine SSRF, but may present some advantages for equine neonatal SSRF (12). For locking plates, the screw is placed at a fixed angle to the plate, increasing the stability of the construct. A unique, veterinary-specific locking plate, the string of pearls (SOP) plate is composed of spherical nodes (pearls) connected by cylindrical internodes, allowing the plate to be contoured in three dimensions, unlike traditional locking plates. The SOP plate has been shown to have greater bending stiffness compared to similar-sized locking plates, which may make it a more ideal implant for some rib fracture configurations (13, 14). In addition, the SOP plate uses cortical, rather than locking screws, by locking the proximal shaft of the screw rather than the screw head. Cortical screws provide slightly greater bone purchase than locking screws because there is a greater difference between the screw’s outer thread and inner shaft, which may make it more desirable in soft, immature bone. Despite these potential advantages, SOP plates have not been evaluated for equine SSRF.

Nylon cable ties (NCT), represent a simplified adaptation of encircling fracture fixation, previously performed with cerclage wire or suture, and is a common reported method for equine neonatal SSRF (9, 11, 22, 23). NCT fixation can be applied rapidly using inexpensive materials with no specialized equipment needed, but the construct may provide greater flexibility to the repaired construct causing greater motion at the fracture site and increasing chances of implant failure. Similarly, clip fixators consist of titanium clips that wrap around the rib using a specific crimping instrument, providing rapid yet flexible rib stabilization with minimal soft tissue disruption. Clip fixators (rib clips, RC) have been investigated and used successfully for human SSRF (8), but their use in veterinary species SSRF has not been reported.

To date, no study has compared the biomechanics of different fixation methods for equine neonatal SSRF. The objective of this study was to evaluate the biomechanical properties of intact and repaired equine neonatal ribs using multiple methods of fixation. We hypothesized that rigid fixation (modified reconstruction plate and SOP) would result in stronger and stiffer repair constructs than non-rigid fixation (NCT and RC). Additionally, we hypothesized that the nylon cable tie and rib clip would fail most commonly at the original fracture.

## 2 Materials and methods

### 2.1 Study design

Neonatal foals submitted for diagnostic postmortem examination to the University of Kentucky Veterinary Diagnostic Laboratory for reasons other than thoracic trauma were used for the study. Foals were included if they were less than 2 weeks of age. Specimens were excluded if, on necropsy at University of Kentucky Veterinary Diagnostic Laboratory, there was evidence of thoracic trauma acquired during birth or thereafter. Signalment and total bodyweight were recorded for each specimen, as well as reasons for euthanasia or death. Both the left and right rib cages were harvested, wrapped in moistened towels, and stored at −80 °C until testing. For each thorax, bilateral ribs 3 to 6 ribs were selected for fixation due to the current recommendation of SSRF in this cranial thoracic region. Sample size was obtained by performing *a priori* power analysis using G*Power (Heinrich-Heine-Universität Düsseldorf, Düsseldorf, Germany). A significance threshold (*α*) of 0.05 and power (1−ß) of 0.80 were assumed, as well as 2-sided tests. A paired *t*-test was used as a surrogate for the linear mixed model analysis and scaled up for 4 groups. A mean increase from control of 100 N for yield load with a standard deviation of 189, and a low correlation (*r* = 0.4) extrapolated from human data resulted in a sample size of 20 ribs/group requiring a total of 10 foal cadavers. Each rib was randomly allocated to one of four repair groups: 4 mm Nylon Cable tie (industrial grade polyamide 6.6) (NCT), a modified 2.7 mm AO reconstruction plate manually reamed to accommodate 3.5 cortical screws referred to as a modified reconstruction plate (Vet ovation Raleigh, North Carolina) (MRP), 2.7 mm string of pearls locking plate (Vet ovation Raleigh, North Carolina) (SOP), and STRACOS™ Titanium 3D rib clip (MedX- pert GmbH, Eschbach, Germany) (RC). Due to the number of variables including ribs tested, side (L or R) of thorax, and repair group, equal distribution of all variables within each horse was not possible. Each rib, considering the side of the thorax from which it was obtained, was randomly allocated to ensure equal representation of all variables within each repair group.

### 2.2 Biomechanical testing and fracture of intact ribs

**Figure 1:**
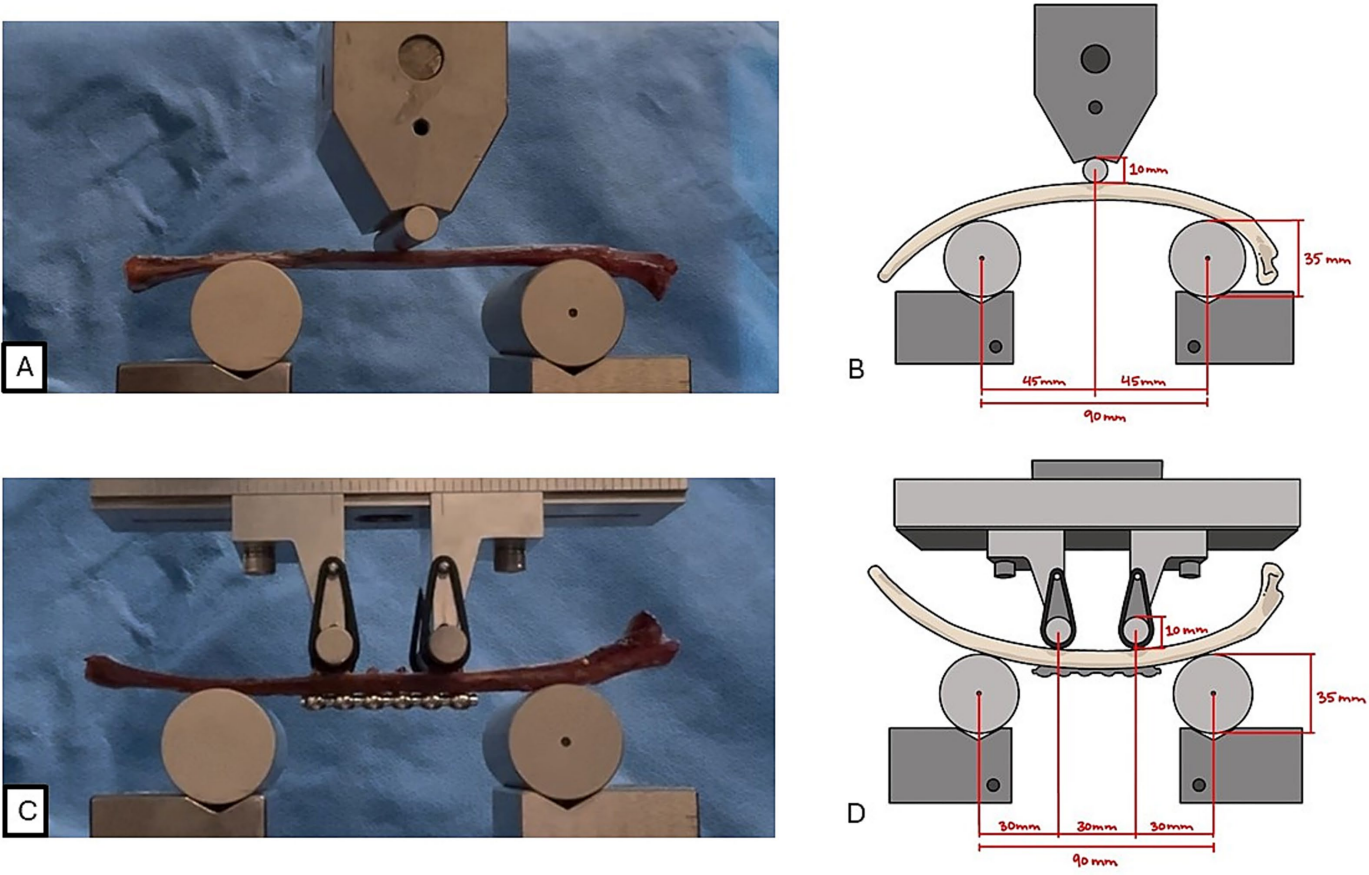
Biomechanical three-point testing set up to fracture intact ribs **(A,B)** and four-point testing set up to test repaired ribs **(C,D)**. A string of pearls (SOP) plate for repair is shown for testing.

At the time of testing, each rib cage was thawed overnight at room temperature. Ribs 3–6 were disarticulated from the vertebrae, and all surrounding soft tissue, including the intercostal musculature, was removed. Measurements of length, height, width, and circumference of each rib were obtained and recorded by the same investigator (KN). Each intact rib was subjected to a single-cycle to failure three-point bending test. The force was applied to the extra-thoracic, concave surface of the rib to simulate external trauma that would be sustained during dystocia, creating a simple, transverse fracture at approximately 50% of the rib length. For all three-point bending tests, the force was applied with a crosshead speed of 0.25 mm/s at a loading distance of 35 mm and support span distance of 90 mm using the Instron 5,565 universal materials testing machine (Instron, Norwood, Massachusetts) (Figures 1A,B). The support span and loading distance were selected to suit all size variations of the tested rib numbers and cadavers. All specimens were loaded to failure, and the maximum force reached (F_max_) and force at failure (F_fail_) recorded.

### 2.3 Repair of fractured ribs

**Figure 2:**
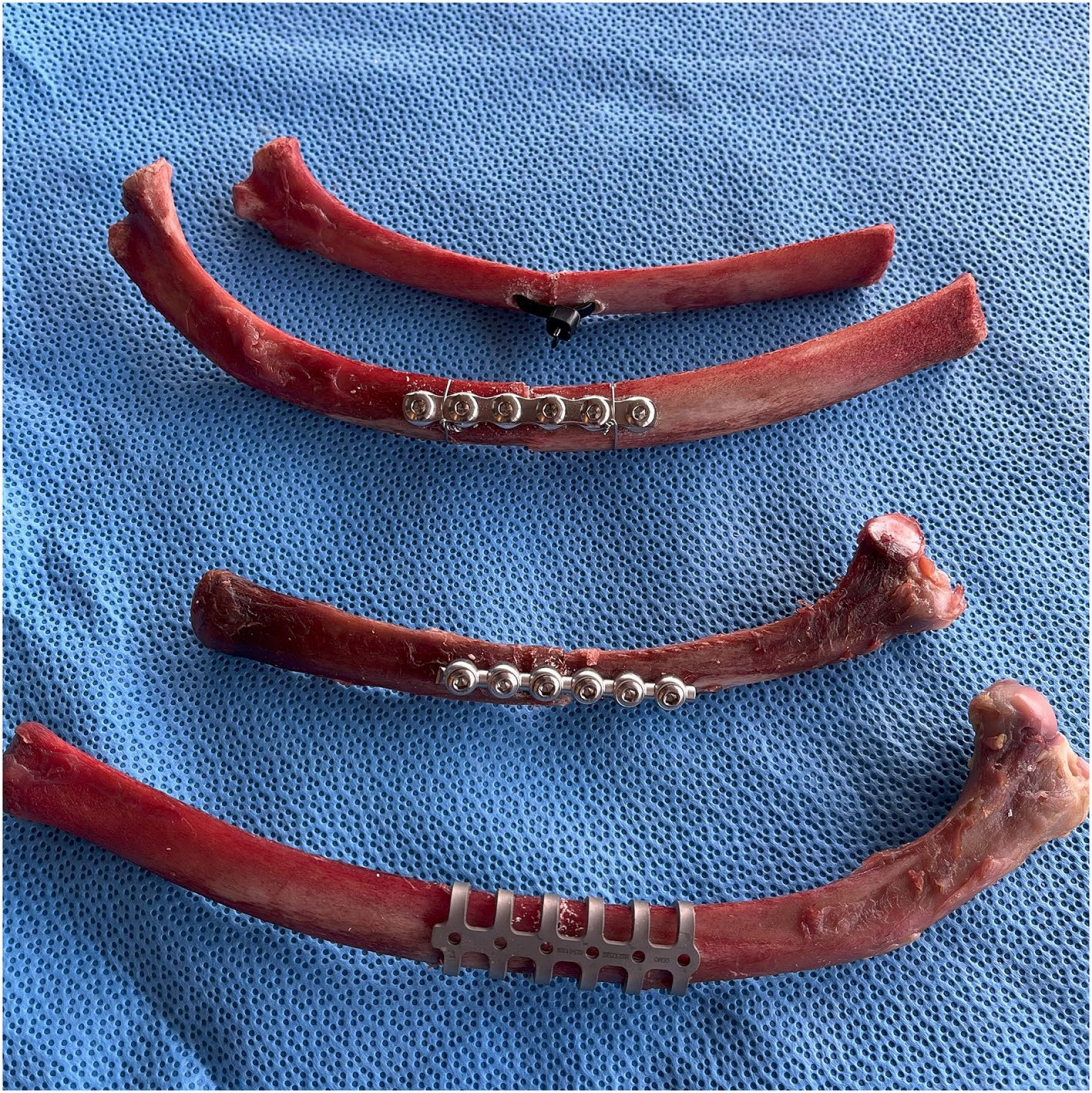
Resected ribs that have been fractured and then repaired using the following methods described from top to bottom: 4 mm nylon cable tie (NCT), 2.7 mm reconstruction plate modified to accommodate 3.2 cortical screws with cerclage wire applied (MRP), 2.7 mm string of pearls veterinary locking plate (SOP) and STRACOS™ Titanium 3D rib clip (RC).

Ribs were then repaired according to their assigned repair group. All repairs were performed by the same investigator (KN). Figure 2 shows an example of the final repaired construct for each group.

For the NCT repair group, a 3.2 mm drill bit was used to drill a central hole 5 mm from the fracture line for both the proximal and distal fragment. Then a 4 mm wide, 406 mm long nylon cable tie was threaded through the drill holes in a parallel orientation to the rib and manually tightened opposing the fracture fragments and reducing the fracture. The excess cable tie was removed close to the lock.

For the modified reconstruction plate (MRP) repair group, a 2.7 mm 26-hole AO reconstruction plate was manually cut into 20, 6-hole plates and then manually reamed with a 3.2 mm bit to accommodate 3.5 mm cortical screws as previously described (15). The fracture was reduced; the plate was contoured if needed, and a bi-cortical thread hole was drilled using the 2.5 mm drill bit through each plate hole. Six, 3.5 mm cortical self-tapping screws, 12 mm in length, were placed and tightened by hand. Two pieces 5 USP cerclage wire was then placed around the rib and plate, proximal and distal to the fracture line, in between the first and second screws and fifth and sixth screws. The wire was tightened using orthopedic wire twisters with excess wire removed.

For the SOP repair group, a 2.7 mm 20-hole SOP plate was manually cut into 20, 6-pearl plates. To repair the fracture, the fracture was reduced, the plate was contoured if needed, and a bicortical thread hole was drilled in the center of the pearl using a 2.0 mm drill bit with a 2.7 mm drill guide. Six, 2.7 mm cortical self-tapping screws 12 mm in length were placed into each drill hole and tightened manually.

For the RC repair group, STRACOS™ Titanium 3D rib clips (Medxpert, Deutschland, Germany) 21 mm in width were cut into 20, 6-prong segments. The fracture was reduced, and the clip was applied using the universal rib clip fixation plier with 3 prongs encompassing the proximal fragment and 3 prongs encompassing the distal fragment. The pliers were placed vertically to the clip, ensuring proper seating of the pliers in the centering hole, and then the clip was tightened to secure the lateral prongs around the rib.

### 2.4 Biomechanical testing of repaired ribs

Repaired ribs underwent a single-cycle to failure four-point bending test using the Instron 5,565 universal materials testing machine (Instron, Norwood, Massachusetts). Force was applied to the intra-thoracic, convex surface of the rib simulating internal stresses that leads to fatigue or failure of the repair at a crosshead speed of 0.25 mm/s and center span distance of 30 mm, with a loading span of 90 mm (Figures 1C,D). F_max_ and F_fail_ of the repaired ribs were recorded. From the load–displacement curves, the stiffness (K, N/mm) was determined by calculating the slope of the linear elastic portion of the curve. Mode of failure was recorded and defined as failure at the original fracture site, screw or implant pull out, new fracture dorsal to the implant, new fracture ventral to the implant, or other fracture.

### 2.5 Statistical analysis

Means and standard deviations were calculated by repair group for F_max_, F_fail_, and stiffness for both intact and repaired ribs. To account for repeated measurements per horse, linear mixed models were used to compare treatment means assuming a compound symmetric covariance matrix. All models adjusted treatment means for rib number (treated as a categorical variable), weight, age, and rib side (left or right). All distributional assumptions were accessed using normality probability plots and histograms. The graphs did not indicate violations of assumptions. If the linear mixed models indicated statistically significant treatment differences, post-hoc comparisons were conducted using a Bonferroni correction. All analyses used a Type I error rate of 0.05 and were programmed in SAS® 9.4. Descriptive statistics were used to report data for rib dimensions and mode of failure.

## 3 Results

In total, 80 ribs underwent three-point testing to fracture the rib and 4-point testing to test each method of repair (20 ribs per group). Fifteen cadavers were needed as some ribs were not intact due to method of harvest and there was a freezer malfunction which would have resulted in a subset of thoraces undergoing an extra freeze–thaw cycle. Due to concerns for differences in tissue handling, these thoraces were excluded from further testing. To ensure appropriate representation of rib # and side of thorax (L or R) additional thoraces were needed. The cadaver study population, therefore, consisted of 14 thoroughbreds and 1 Gypsy Vanner. The median age was 2 days old (mean 2.7 ± 3.8 days) with a mean body weight of 130.8 ± 21.7 lbs. The mean rib dimensions were length 185.71 ± 25.6 mm, width 15.56 ± 14.73 mm, height 6.18 ± 0.70 mm, and circumference 36.67 ± 2.50.

**Figure 3:**
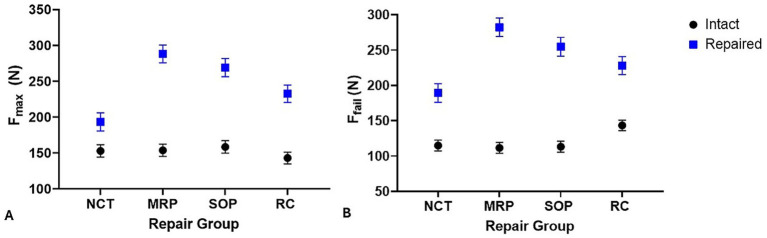
F_max_ **(A)** and F_fail_ **(B)** for intact and repaired ribs for each repair group.

For intact ribs, there was no difference in F_max_ (*p* = 0.46) or F_fail_ (*p* = 0.35) among repair groups. For repaired ribs, there were differences in F_max_ (*p* < 0.0001) and F_fail_ (*p* < 0.0001) between groups (Table 1). Multiple comparison analysis with a Bonferroni correction identified differences among repair groups for both F_max_ and F_fail_. Fracture repair with NCT produced significantly lower Fmax and F_fail_ compared to MRP, SOP, and RC. The Fmax and F_fail_ were significantly higher for MRP compared to RC repair, but similar to Fmax and Ffail for SOP repair. The Fmax and Ffail for RC repair were significantly higher than NCT, but significantly lower than MRP (Figures 3A,B).

**Table 1:** Mean ± standard error for F_max_ (N), F_fail_ (N), and stiffness (N/mm) intact and repaired ribs.

Outcome Variable	Repair group				*P*-value
	Nylon cable tie	Modified reconstruction plate	String of pearls plate	Rib clip	
	(Mean ± SEM)				
F_max_ (N)					
Intact	152.98 ± 8.62	153.87 ± 8.49	158.54 ± 8.70	143.04 ± 8.31	0.4617
Repaired	193.31 ± 12.65^2,3^	288.18 ± 12.45^1,4^	269.02 ± 12.77^1^	232.64 ± 12.19^2^	<0.0001
F_fail_ (N)					
Intact	115.01 ± 7.67	111.69 ± 7.59	113.28 ± 7.72	143.48 ± 7.41	0.3509
Repaired	189.55 ± 13.20^2,3^	282.36 ± 12.99^1,4^	254.83 ± 13.33^1^	228.25 ± 12.74^2^	<0.0001
Stiffness (N/mm)					
Intact	37.90 ± 3.31	38.50 ± 3.26	39.29 ± 3.35^2^	40.82 ± 3.20	0.8967
Repaired	34.12 ± 3.53^2,3^	78.93 ± 3.49^1,4^	78.98 ± 3.57^1,4^	31.28 ± 3.44^2,3^	<0.0001

Superscripts indicate the column or repair group (1 = NCT, 2 = MRP, 3 = SOP, 4 = RC) that is different from the referenced value within each row using Bonferroni correction.

**Figure 4:**
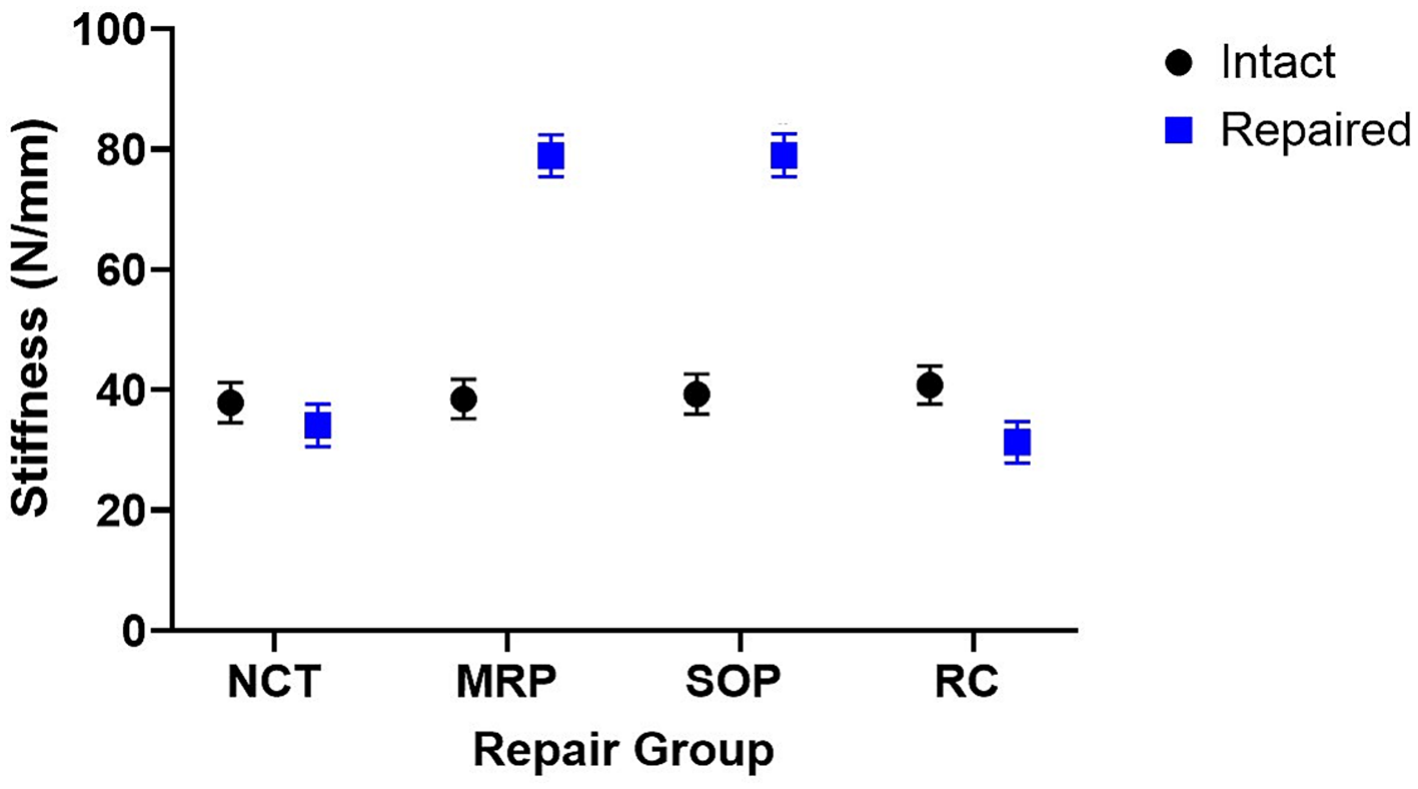
Stiffness (N/mm) for intact and repaired ribs for each repair group.

For intact ribs, there was no difference in stiffness between groups (*p* = 0.8967). For repaired groups, differences in stiffness between groups were observed (*p* < 0.0001). Comparison analyses indicate higher stiffness in constructs repaired with the MRP and SOP than both NCT and RC. All remaining comparisons for stiffness were non-significant (Figure 4).

The mode of failure was different between constructs. The NCT failed at the original fracture (11/20, 55%) or failed through a new fracture that propagated through the dorsal hole in which the NCT was coursing through (9/20, 45%). Screw pull-out occurred most in the MRP (7/20, 35%) and SOP (12/20, 60%) groups. Other modes of failure identified for MRP repair were, new fracture at the dorsal aspect of the implant (6/20, 30%), new fracture at the ventral aspect of the implant (2/20, 10%), and other new fractures (4/20, 20%). Other modes of failure for SOP were a new fracture at the dorsal aspect of the implant (3/20, 15%), a new fracture at the ventral aspect of the implant (2/20, 10%), and other new fractures (3/20, 15%). RC repair failed only at the original fracture line (20/20, 100%).

## 4 Discussion

This is the first *ex vivo* study to evaluate repair strength of four different fixation methods for equine neonatal rib fracture. Consistent with our hypothesis, rigid fixation methods (MRP and SOP) produced stronger and stiffer constructs than non-rigid fixation (NCT and RC). Failure modes also differed between fixation types with non-rigid constructs (NCT and RC) most commonly failing at the original fracture site, whereas SOP constructs primarily failed by screw pullout, and MRP exhibited variable failure modes.

All repair constructs demonstrated greater strength than intact ribs; although stiffness varied considerably between fixation methods. MRP, SOP, and RC constructs were stiffer than intact ribs, whereas NCT constructs remained comparable in stiffness to intact ribs. These findings contrast with those of a recent human rib biomechanical study, in which all constructs were less strong and less stiff than intact, non-fixated rib (16). Prins et al. (16) used ribs obtained from aged adult cadavers, while our study evaluated skeletally immature bone obtained from neonatal foals less than 2 weeks of age. Few studies have investigated the biomechanical behavior of immature ribs. Immature bone has been shown to be more porous and elastic, which means it can sustain higher loads to failure than mature bone (17, 18). In addition, there can be extreme variation in microstructure throughout the rib during growth leading to variable strength and stiffness (17). Intact foal ribs in our study exhibited slightly higher strength and stiffness than immature porcine ribs up to 6 weeks of age indicating that some differences may be species related (19). Optimal rib repair strength and stiffness are not well defined, but ideally, repair strength and stiffness should approximate that of intact bone while preserving rib cage motion to support respiratory mechanics. Repairs that are stiffer than naive bone can cause stress-shielding or stress concentration at the bone-implant interface, impairing fracture healing or causing implant failure (20). In the study by Prins et al., plate and screw systems demonstrated the greatest stiffness, while clamping systems had lower stiffness. In our study, the NCT and RC constructs had lower stiffness than intact ribs, demonstrating the flexible, less rigid nature of these repair methods, which may be advantageous for certain rib fractures. The ideal repair should also be tailored to fracture characteristics (chronicity, configuration, number of ribs involved) and bone properties (immature versus mature or osteoporotic) to minimize hardware failure and implant-related complications.

Implant-related complications remain an important consideration in equine neonatal SSRF. Reported complications include implant migration, construct failure and screw loosening (15, 21–23). In the present study, rigid fixation constructs most commonly failed at the bone-implant interface, whereas non-rigid constructs failed at the fracture site itself. These differing failure patterns may influence implant selection depending on fracture characteristics.

The RC has been designed for the human adult ribs, which differ in size and shape from the equine neonatal ribs, therefore, the clamps did not fully contact the bone, instead they encircled the rib in a cage-like configuration with minimal bone-clamp contact. This likely contributed to reduced strength and stiffness in our study. Design modifications tailored to equine neonatal ribs could improve performance, though cost considerations would need to be addressed for the veterinary market. Both the MRP and the SOP were applied using cortical screws of the same length, engaging both cortices of the rib. The comparable performance of MRP and SOP constructs was notable. Although, SOP plates are designed to provide increased construct stability due the fixed angle of fixation, superior strength was not observed in the present study. While both constructs used bi-cortical screw placement, the SOP construct used 2.7 mm cortical screws and cerclage wire was not used to augment the repair, differences that may have impacted the results when the SOP was compared to the MRP. When securing plates with screws, bi-cortical engagement is recommended for compression plating but is not necessary for locking plate constructs. A recent study in human SSRF comparing uni-cortical versus bi-cortical screw placement found no difference in construct strength or failure. In the present study, ribs were repaired with screws engaging both cortices of the bone. While descriptions in the literature are not always explicit, reported screw lengths suggest that most equine rib repairs employ bi-cortical placement. Care must be taken to avoid excessively long screws, which can damage underlying structures when placed bi-cortically. Further work to investigate uni-cortical screw placement in both reconstruction and locking plate systems to determine its effect on construct strength and failure modes in immature bone may be valuable.

Long-term outcome data for foals with rib fractures is largely limited to racing performance. Fehin et al. (24) evaluated 44 foals with rib fractures, reporting 11 foals surviving that were treated surgically and 34 foals surviving treated medically. Foals undergoing SSRF had similar return-to-racing and median earnings compared to those treated conservatively (25). Velloso et al. (15) assessed 73 neonates undergoing fracture repair of 1 or more ribs, comparing outcomes to their non-fractured maternal siblings. Repairs included external splints secured percutaneously with suture or cerclage wire, reconstruction plates with screws or cerclage wire, or nylon cable ties. The method of repair did not affect outcomes, with 78% of surgically treated foals surviving to discharge and 61% of these foals started a race (15). These retrospective studies indicate that foals can survive and return to work after rib fracture repair. However, long-term data on tack-related comfort, behavior under saddle, or musculoskeletal health, including lameness, remain lacking.

This study has several limitations. Stiffness and strength of intact ribs were assessed using three-point bending because preliminary testing showed that neonate ribs were too flexible for four-point bending, with ribs bending around both top cylinders rather than fracturing along the rib. Three-point bending was chosen over standard ostectomy to evaluate the inherent biomechanical properties of naïve, intact ribs. Repaired ribs, however, were tested using four-point bending in a different orientation (extra- to intrathoracic versus intra- to extra-thoracic), preventing direct comparison with intact ribs. Fracture location differed from naturally occurring neonatal fractures to permit standardized mechanical testing. The fracture location was chosen to ensure symmetrical placement in the Instron testing machine and prevent uneven application of load. Ribs were tested under bending rather than compression to more accurately reflect lateral forces believed to cause neonatal rib fractures. Cyclic loading, which could provide more information regarding implant fatigue compared to implant failure, was not performed and should be used for testing in future studies to more accurately reflect physiologic conditions.

Furthermore, testing was performed in isolated ribs under quasi-static loading, without adjacent ribs or supporting soft tissues. *In situ*, fractured ribs would experience dynamic forces distributed across the rib cage, which could alter biomechanical behavior. Implants did not undergo sterilization prior to application and testing the effect of different methods of sterilization for some of the implants used has been previously evaluated and should be considered prior to use (26). Future studies should evaluate cyclic loading, construct augmentation and screw configurations as well as *in situ* changes to respiratory mechanics.

Despite these limitations, this study is the first to characterize the biomechanical properties of intact equine neonatal ribs and to evaluate commonly used repair techniques, providing a foundation for further investigation and optimization of repair methods.

## 5 Conclusion

In conclusion, current repair methods significantly enhance the strength of fractured equine neonatal ribs compared to naïve ribs. Repair with MRP, SOP and RC constructs also provided increased stiffness compared to intact ribs. Selection of the appropriate SSRF construct should be guided by fracture characteristics, degree of displacement, patient condition, and the clinician’s experience and available equipment. Further research is needed to define the optimal biomechanical properties of repair constructs for equine neonates and to evaluate their impact on respiratory function, fracture healing, implant durability, and long-term patient outcomes, ultimately informing best practices for the management of neonatal equine rib fractures.

## Data Availability

The raw data supporting the conclusions of this article will be made available by the authors, without undue reservation.

## References

[1] Harrison L. Equine fracture cases. Equine Dis Quart. (1995) 3

[2] Jean D Laverty S Halley J Hannigan D Leveille R. Thoracic trauma in newborn foals. Equine Vet J. (1999) 31:149–52. doi: 10.1111/j.2042-3306.1999.tb03808.x, 10213427

[3] Sprayberry KA Bain FT Seahorn TL Slovis NM Byars TD editors. Cases of rib fractures in neonatal foals hospitalized in a referral center intensive care unit from 1997–2001. Proceedings American Association of Equine Practitioners; (2001) 47, 395–399.

[4] Jean D Picandet V Macieira S Beauregard G D'Anjou MA Beauchamp G. Detection of rib trauma in newborn foals in an equine critical care unit: a comparison of ultrasonography, radiography and physical examination. Equine Vet J. (2007) 39:158–63. doi: 10.2746/042516407x166657 17378445

[5] Bauman ZM Tian Y Doben AR Schubl SD Pieracci FM Kaye AJ . Chest wall injury society guidelines for surgical stabilization of rib fractures: indications, contraindications, and timing. J Trauma Acute Care Surg. (2025) 99:522–32. doi: 10.1097/ta.0000000000004750, 40802491 PMC12893141

[6] Prins JTH Wijffels MME Pieracci FM. What is the optimal timing to perform surgical stabilization of rib fractures? J Thorac Dis. (2021) 13:S13–s25. doi: 10.21037/jtd-21-649, 34447588 PMC8371546

[7] Worn M Jones MD. Rib fractures in infancy: establishing the mechanisms of cause from the injuries—a literature review. Med Sci Law. (2007) 47:200–12. doi: 10.1258/rsmmsl.47.3.200, 17725233

[8] Joseph N McGuinness MJ Ratnayake M Wells C Phillips A Cornish J . Current and emerging techniques for surgical stabilisation of rib fractures: a systematic scoping review. ANZ J Surg. (2026) 96:1073–85. doi: 10.1111/ans.70464, 41578721

[9] Kraus BM Richardson DW Sheridan G Wilkins PA. Multiple rib fracture in a neonatal foal using a nylon strand suture repair technique. Vet Surg. (2005) 34:399–404. doi: 10.1111/j.1532-950X.2005.00061.x, 16212597

[10] Bellezzo F Hunt RJ Provost P Bain FT Kirker-Head C. Surgical repair of rib fractures in 14 neonatal foals: case selection, surgical technique and results. Equine Vet J. (2004) 36:557–62. doi: 10.2746/0425164044864561, 15581318

[11] Williams TB Williams JM Rodgerson DH. Internal fixation of fractured ribs in neonatal foals with nylon cable tie using a modified technique. Can Vet J. (2017) 58:579–81. 28588328 PMC5432144

[12] Hall S Smith R Ramzan PHL Head M Robinson N Parker R. Rib fractures in adult horses as a cause of poor performance; diagnosis, treatment and outcome in 73 horses. Equine Vet J. (2023) 55:59–65. doi: 10.1111/evj.13566, 35170087

[13] Ness M. The effect of bending and twisting on the stiffness and strength of the 3.5 SOP implant. Vet Comp Orthop Traumatol. (2009) 22:132–6. doi: 10.3415/vcot-08-03-0030, 19290394

[14] DeTora M Kraus K. Mechanical testing of 3.5 mm locking and non-locking bone plates. Vet Comp Orthop Traumatol. (2008) 21:318–22. doi: 10.3415/VCOT-07-04-0034, 18704237

[15] Velloso Álvarez A Sandow CB Rodgerson DH Spirito MA. Survival and racing performance after surgical treatment of rib fractures in foals. Vet Surg. (2022) 51:62–7. doi: 10.1111/vsu.13701, 34486743

[16] Prins JTH Van Wijck SFM Leeflang SA Kleinrensink G-J Lottenberg L De La Santa Barajas PM . Biomechanical characteristics of rib fracture fixation systems. Clin Biomech (Bristol, Avon). (2023) 102:105870. doi: 10.1016/j.clinbiomech.2023.105870, 36623327

[17] Agnew AM Moorhouse K Kang Y-S Donnelly BR Pfefferle K Manning AX . The response of Pediatric ribs to quasi-static loading: mechanical properties and microstructure. Ann Biomed Eng. (2013) 41:2501–14. doi: 10.1007/s10439-013-0875-6, 23907336

[18] Agnew AM Schafman M Moorhouse K White SE Kang Y-S. The effect of age on the structural properties of human ribs. J Mech Behav Biomed Mater. (2015) 41:302–14. doi: 10.1016/j.jmbbm.2014.09.002, 25260951

[19] Beadle N Burnett TL Hoyland JA Sherratt MJ Freemont AJ. A novel ex vivo model of compressive immature rib fractures at pathophysiological rates of loading. J Mech Behav Biomed Mater. (2015) 51:154–62. doi: 10.1016/j.jmbbm.2015.06.031, 26253206

[20] Chmielewska A Dean D. The role of stiffness-matching in avoiding stress shielding-induced bone loss and stress concentration-induced skeletal reconstruction device failure. Acta Biomater. (2024) 173:51–65. doi: 10.1016/j.actbio.2023.11.011, 37972883

[21] Garrett KS Embertson RM Hopper SA Woodie JB McQuerry KJ. Preoperative computed tomographic evaluation of neonatal foals with rib fractures. Vet Surg. (2022) 51:816–26. doi: 10.1111/vsu.13817, 35500138

[22] Downs C Rodgerson D. The use of nylon cable ties to repair rib fractures in neonatal foals. Proceedings of the American Association of Equine Practitioners (2008) 53:64–67.

[23] Downs C Rodgerson DH. How to Repair Fractured ribs in Neonatal Foals using Nylon Cable Ties. (2008).

[24] Fehin WF Wylie CE Feeney C Perez Olmos JF Marr CM. The future racing performance of neonatal thoroughbreds diagnosed with rib fractures treated both surgically and conservatively. Equine Vet J. (2017) 49:5–29.

[25] Thoracic UGTRA. The future racing performance of neonatal thoroughbreds diagnosed with rib fractures treated both surgically and conservatively. Equine Vet J. (2017) 49:5–29.

[26] Sicard GK Hayashi K Manley PA. Evaluation of 5 types of fishing material, 2 sterilization methods, and a crimp-clamp system for extra-articular stabilization of the canine stifle joint. Vet Surg. (2002) 31:78–84. doi: 10.1053/jvet.2002.30539, 11778171

[27] Schambourg MA Laverty S Mullim S Fogarty UM Halley J. Thoracic trauma in foals: post mortem findings. Equine Vet J. (2003) 35:78–81. doi: 10.2746/042516403775467478, 12553467

[28] Sprayberry KA Barrett EJ. Thoracic trauma in horses. Vet Clin North Am Equine Pract. (2015) 31:199–219. doi: 10.1016/j.cveq.2014.12.001, 25770070

[29] Polycarpou A Kim BD. Thoracic trauma in horses. Vet Clin North Am Equine Pract. (2021) 91:947–950. doi: 10.1097/TA.0000000000003376, 25770070

[30] Sarani B Allen F Pieracci FM Doben AR Eriksson E Bauman ZM . Characteristics of hardware failure in patients undergoing surgical stabilization of rib fractures: A chest wall injury society multicenter study. J Trauma Acute Surg. (2019) 87:1277–1281. doi: 10.1097/ta.0000000000002373, 31107433

